# Damage to the Fronto-Polar Cortex Is Associated with Impaired Multitasking

**DOI:** 10.1371/journal.pone.0003227

**Published:** 2008-09-16

**Authors:** Jean-Claude Dreher, Etienne Koechlin, Michael Tierney, Jordan Grafman

**Affiliations:** Cognitive Neuroscience Section, National Institute of Neurological Disorder and Stroke, National Institutes of Health, Bethesda, Maryland, United States of America; University of California San Diego, United States of America

## Abstract

**Background:**

A major question in understanding the functional organization of the brain is to delineate the functional divisions of the prefrontal cortex. Of particular importance to the cognitive capacities that are uniquely human is the fronto-polar cortex (Brodmann's area 10), which is disproportionally larger in humans relative to the rest of the brain than it is in the ape's brain. The specific function of this brain region remains poorly understood, but recent neuroimaging studies have proposed that it may hold goals in mind while exploring and processing secondary goals.

**Principal Findings:**

Here we show that the extent of damage to the fronto-polar cortex predicts impairment in the management of multiple goals. This result reveals that the integrity of the fronto-polar cortex is necessary to perform tasks that require subjects to maintain a primary goal in mind while processing secondary goals, an ability which is crucial for complex human cognitive abilities.

**Conclusion/Significance:**

These results provide important new insights concerning the cerebral basis of complex human cognition such as planning and multitasking.

## Introduction

Some of the most complex cognitive abilities of humans, such as planning, are commonly attributed to a disproportionate enlargement of the human frontal lobe during evolution. However, recent comparative studies of the relative size of the frontal cortex taken as a whole indicate that the human frontal cortex is not larger in comparison to those of the great apes [1], [2]. Rather, the specific cognitive capacities of humans may be due to differences in specific individual cortical areas (such as the frontopolar cortex), as well as to richer interconnectivity between the frontal lobe and other higher-order association areas, none of which require an increase in the overall relative size of the frontal lobe during hominid evolution.

Of particular importance in the cognitive capacities that are uniquely human seems to be the most anterior part of the prefrontal cortex, namely the fronto-polar cortex (Brodmann's area 10), which is larger in humans relative to the rest of the brain than it is in the ape's brain [1], [2]. The specific function of this brain region remains poorly understood, but one recent hypothesis states that its role is to hold goals in mind while exploring and processing secondary goals [3], [4], [5], [6] – a process that we refer to as multitasking in the remainder of this paper-. Neither keeping in mind a goal over time (working memory) nor successively allocating attentional resources between alternative goals (dual-task performance) could by themselves selectively activate the fronto-polar cortex while a highly specific super-additive effect was demonstrated in the frontopolar cortex when subjects held in mind goals while processing secondary goals at the same time [3].

This functional hypothesis about a key role of Brodmann's area (BA) 10 in multitasking is based on the results of functional neuroimaging studies that can only support inferences about the association of brain regions with a specific cognitive process. In contrast, neuropsychological studies are crucial for inferring whether a brain region is necessary to mediate a cognitive process.

Here we examined patients with focal prefrontal cortex lesions to test whether the fronto-polar cortex is necessary for multitasking. Our hypothesis was that the extent of damage to the fronto-polar cortex should correlate with impairment in this process. The results confirmed this hypothesis by demonstrating that the extent of damage to Brodmann's area 10 correlated with impaired multitasking.

## Materials and Methods

### Participants

**Figure 1:**
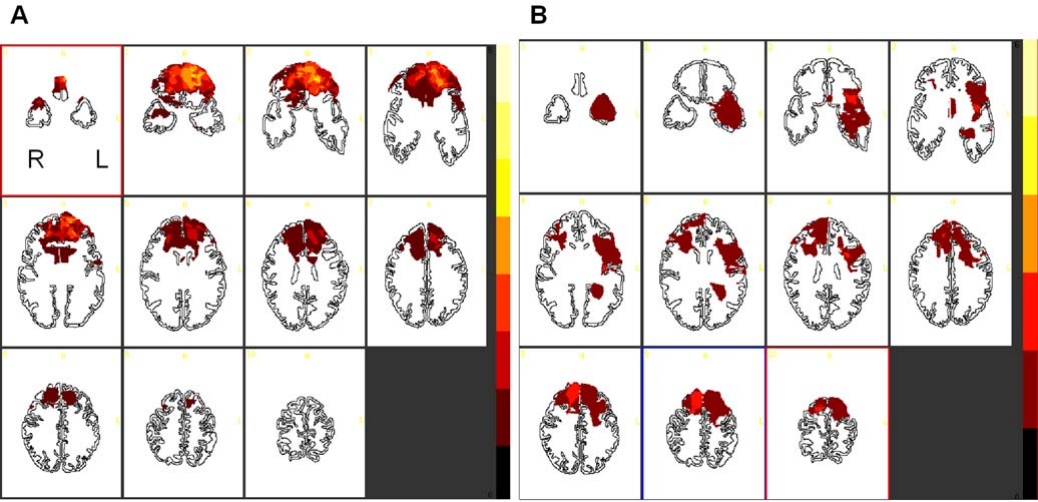
(A). Location and degree of lesion overlap in patients with fronto-polar cortex lesions. (B). Location and degree of lesion overlap in control patients without fronto-polar cortex lesions. Slices are oriented in radiological convention (i.e. the left side of the image is the right hemisphere). Lighter colors denote the degree to which lesions involve the same structure in multiple subjects. The darker color at the bottom of the color scale indicates no overlap between brain region.

We tested 13 patients with focal frontal lobe lesions (see **Table 1** and **Figs 1** **–** [Fig pone-0003227-g002] **3** **for** patient's demographic and lesion's sites). Patients were divided into two groups on the basis of the location of their lesion: one group had lesions that included the fronto-polar cortex (n = 7, 4 males, mean age = 49±6) and one group had lesions that excluded the fronto-polar cortex (n = 5, 3 males, mean age = 49±3.5). The two groups of patients did not differ in age (t = 0.03, P = 0.98), level of education (t = 0.29, P = 0.8) and Full Scale WAISIII IQ scores (t = 0.74, P = 0.48). In addition, 7 control subjects (5 males, mean age = 50.8±6) were matched in age (t = 0.2, P = 0.82, n.s) and level of education (t = 0.72, P = 0.48) with the patients with fronto-polar lesions. All subjects were screened for a prior history of neurological disease, substance abuse, and psychiatric disorder.

**Figure 2 pone-0003227-g002:**
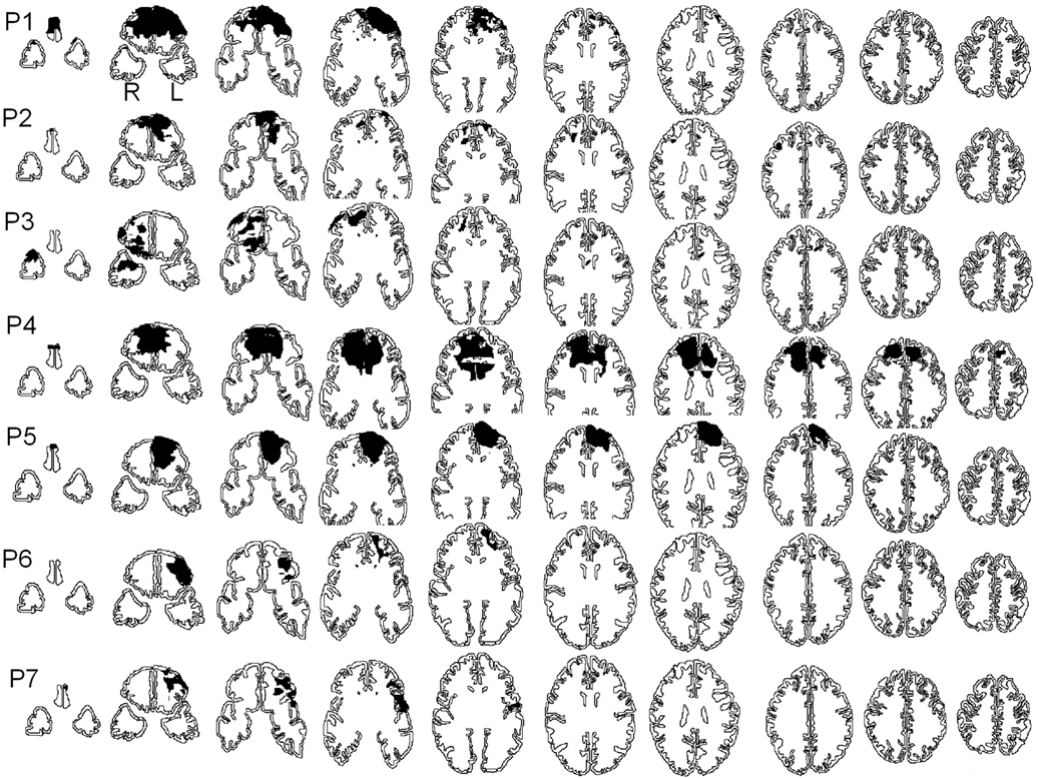
Reconstruction of lesions for each patient with frontopolar cortex damage (P1–P7) based on computerized tomography and MRI scans. The shaded area represents the lesion. Axial slices from ventral (left) to dorsal (right). According to radiological convention right is left.

**Figure 3:**
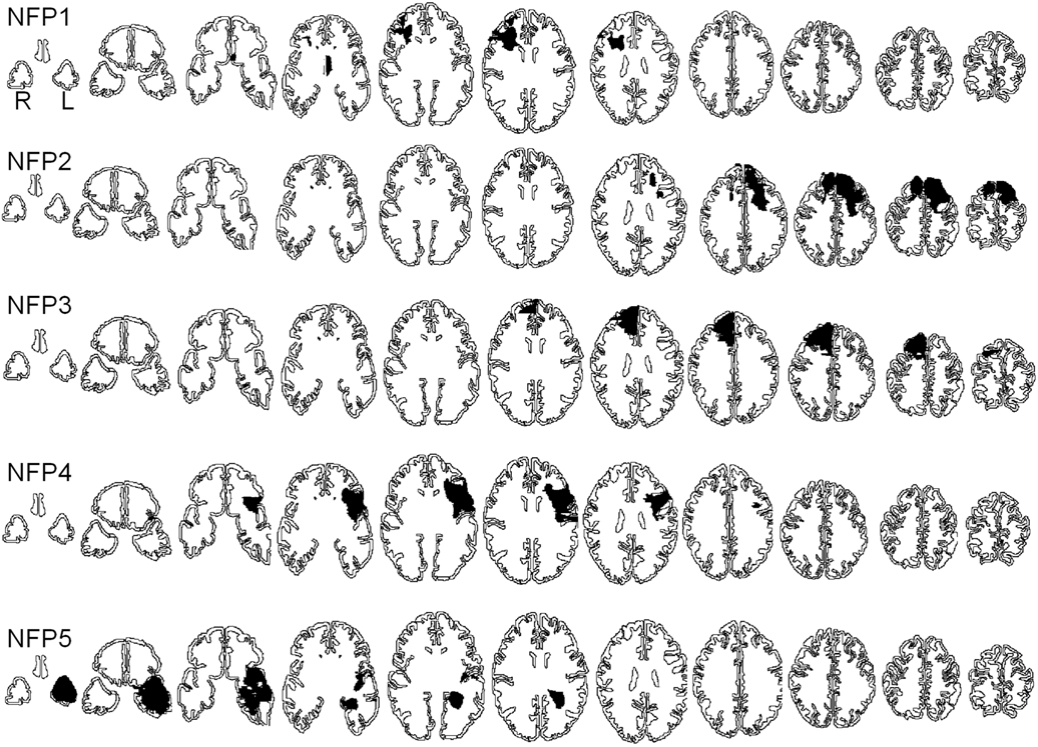
Reconstruction of lesions for each patient without frontopolar cortex damage (NFP1–NFP5) based on computerized tomography and MRI scans. The shaded area represents the lesion. Axial slices are displayed in radiological convention from ventral (left) to dorsal (right).

**Table 1:** Demographic of patients and healthy control subjects.

Group	Age	Level of Educa-tion	Sexr	Hand	Time since lesion (years)	WAIS-III Full Scale IQ	WMS - III Working Memory Index Score	WMS - III Auditory Immediate Index Score	WMS - III Auditory Recognition Delayed Index Score	Fluency Total Raw Score	BNT Total Raw Score	BDI-2 Total Raw Score	NART Full IQ
**FP Lesions**													
FP1	47	11	M	R	31	80	108	89	89	31	34	1	
FP2	62	20	M	R	7	121	88	120	105	74	59	9	
FP3	56	12	M	R	33	102	96	80	100	27	57	3	
FP4	39	14	F	R	13	104	111	114	95	30	56	17	
FP5	28	18	F	R	13	132	124	117	100	77	55	33	
FP6	62	16	F	R	31	137	108	114	110	57	54	3	
FP7	49	12	M	R	39	97	96	65	65	14	52	5	
**No FP lesion**													
NFP1	52	14	M	L	32	99	91	102	90	29	57	19	
NFP2	55	16	F	R	1	101	77	80	95	37	16	14	
NFP3	54	10	M	R	32	98	108	92	105	22	51	3	
NFP4	45	15	M	R	8	118	115	74	85	31	54	7	
NFP5	38	16	F	R	1	100	93	108	110	38	58	3	
**Controls**													
C1	50	13	M	R								0	110
C2	45	12	M	R								NA	NA
C3	63	12	M	R								2	112
C4	62	16	M	L								4	102
C5	55	12	M	R								0	91
C6	50	12	M	R								0	121
C7	28	18	F	R								2	112

The etiology of fronto-polar patients was as followed: 3 were Vietnam veterans who suffered a penetrating brain injury, one patient had a resection of a malignant tumor, one had an aneurysm of the right anterior communicating artery and two patients had closed head injuries. Reconstruction of lesions for each patient with or without frontopolar cortex damage is provided in figures 2 and 3. All subjects were screened for a prior history of neurological disease, substance abuse, and psychiatric disorder. The control subjects were paid for their participation. Subjects provided written informed consent approved by the NINDS Institutional Review Board.

### Lesion analysis

The penetrating brain injury patients were scanned using a standard CT sequence since they had retained metal in their brain. Other patients were scanned by MRI using a three-dimensional set acquisition in the axial plane with a SPGR T1-weighted sequence and a T2-weighted axial sequence. All lesions were traced using the Analysis of Brain Lesions software package [7] and normalized in Talairach space to the Damasio's template [8]. Then, the percentage of the approximate Brodmann areas contained within the boundaries of the lesions was computed for each subject using this standardized, semi-automated software that can determine the extent of brain lesions in terms of cytoarchitectonic regions in Talairach space [7] (see **Fig. 1**).

### Behavioral paradigm

**Figure 4:**
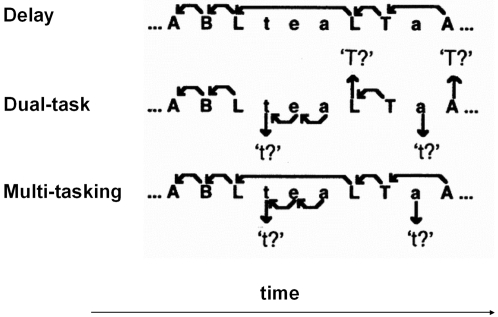
Behavioural tasks. Single-letters (upper or lower-case) from the word “tablet” were successively presented and subject's decisions were recorded using two single response-buttons, one for each hand. Delay condition: subjects decided whether two successively presented upper-case letters were also in immediate succession in the word “tablet” by pressing the right button for yes and the left button if they were not in succession, and they had to ignore lower case letters that were presented in order to delay the response required for upper-case letters. Dual-task condition: subjects decided whether two successively presented letters were also in immediate succession in the word “tablet” by pressing the right button for yes and the left button if they were not in succession, this time both for upper and lower case letters, except that they had to decide whether every first letter indicating a case change was the letter T (or t). Multi-tasking condition: subjects responded to upper case letters exactly as in the delay condition and to lower case letters exactly as in the dual task condition. Thus, the multitasking condition requires maintenance of the primary task information in memory (primary goal) so that it can be returned to after completing a secondary task (subgoal).

The tasks were identical to those used in a previous fMRI study [3] and were designed to systematically vary keeping in mind a main goal over time (working memory) and allocation of attentional resources between alternative subgoals (dual-task) (**Fig. 4**). The experiment consisted of 6 runs in which 3 tasks (delay, dual and multi-tasking, described hereafter) were administered in pseudo-random order (28 trials by task, inter-stimuli interval = 3 s). This pseudo-random order was built in such a way that each condition appears at all serial positions within a run and two conditions appeared once or twice in immediate succession to prevent confounding order effects. The first run was used for training and is not included in the present analysis.

Subjects responded to visually presented letters (500 ms duration, 3000 ms stimulus-onset-asynchrony) by pressing response buttons with their right (match) or left (no match) hand, respectively. Subjects were given standard instructions to respond quickly and accurately. Single-letters (upper or lower-case) from the word “tablet” (i.,e A, B, E, L, T, a, b, e, l, t) were successively presented and subject's decisions were recorded using the two single response-buttons. Matching proportions were maintained between 40 and 43% of trials in each condition. In all conditions lower-case letters were pseudorandomly presented in 64% of trials and the mean SOA between two successive upper-case letters was strictly maintained at 6.3 s. The tasks were administered using the Expe6 software package [9].

In the delay condition, subjects decided whether two successively presented upper-case letters were also in immediate succession in the word “TABLET” by pressing the right button for yes and the left button if they were not in succession, and they had to ignore lower case letters that were presented in order to delay the response required for upper-case letters.

In the dual-task condition, subjects decided whether two successively presented letters were also in immediate succession in the word “tablet” or “TABLET” (this time both for upper and lower case letters), by pressing the right button for yes and the left button if they were not in succession, except that they had to decide whether every first letter indicating a case change was the letter T (or t).

In the multitasking condition, subjects responded to upper case letters exactly as in the delay condition and to lower case letters exactly as in the dual task condition. Thus, the multitasking condition requires maintenance of the primary task information in memory (primary goal) so that it can be returned to after completing a secondary task (subgoal). In other words, for successive upper case letters, or for successive lower case letters, subjects decided whether the current letter followed immediately the previously presented letter in the word “TABLET” or ‘tablet” by pressing the right button for yes and the left button if they were not in succession, and they had to decide whether every first letter indicating a case change was the letter T (or t).

## Results

### Behavioral performance

**Figure 5:**
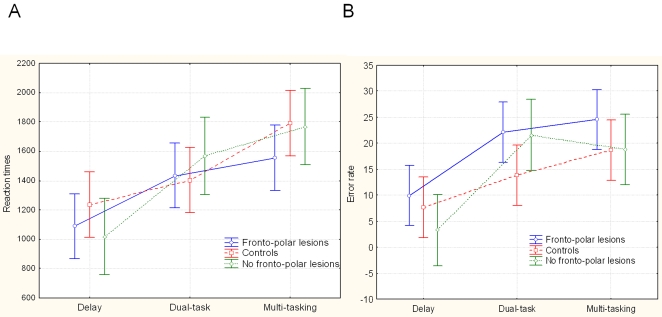
Behavioral performance. (A). Graph representing responses times (for correct responses) in patients with fronto-polar cortex lesions (blue), in patients without fronto-polar cortex lesions (green) and in controls (red). (B). Percentage of errors in the three conditions (delay, dual and multi-tasking) in patients with fronto-polar lesions (blue), in patients without fronto-polar cortex lesions (green) and in normal controls (red). Error bars represent the 95% confidence intervals.

First, a 2*3 repeated measures ANOVA was conducted on correct reaction time (RT) (<3000 ms) and on error rates with group (patients with fronto-polar cortex lesions and age-matched controls) as the between-subject factor and with conditions (delay, dual task, multi-tasking) as the within-subject factor (**Fig. 5**). In this group analysis of variance, for response times, there was a main effect of task [F(2, 36) = 10.6, P<0.0005], indicating that additional processes are engaged successively in the delay, dual-task and multi-tasking conditions. No main effect of group [F(1,36) = 2.0, P = 0.16] and no group*task interaction [F(2,36) = .25, P = .8] were observed.

For error rates, patients with lesions affecting the fronto-polar cortex made more errors than controls (main effect of group [F(1, 36) = 5.01, P<0.05]). There was also a main effect of task [F(2, 36) = 9.8, P<0.0005]. Overall, the multi-tasking condition led to more errors than the delay condition (F(1,26) = 21.5, P<0.0001) but did not differ from the dual-task condition [F(1,26) = 1.2, P = 0.3]. We did not observe any group*task interaction [F(2,36) = 0.5, P = 0.6] on performance accuracy. However, when performing a new 2*2 analysis of variance grouping the dual task and multi-tasking conditions together (both involving putting information into sequences) compared to the delay condition, we found a significant difference between patients with frontopolar lesions and controls [F(1,26) = 5.04, P<0.05]. No between group difference was observed when performing another 2*2 analysis of variance grouping the delay and multi-tasking conditions together (both involving working memory) compared to the dual task condition [F(1,26) = 1.24, P = 0.27].

We also directly compared the performance of the two groups of patients. For response times, there was a main effect of task [F(2,30) = 11.6, P<0.0005], confirming additional engagement of processes from the delay to the dual-task and to the multi-tasking conditions. No main effect of group [F(1,30) = .74, P = 0.39] and no group*task interaction [F(2,30) = .63, P = .53] were observed for response times. For error rates, there was a trend towards significance in patients with lesions affecting the fronto-polar cortex compared to patients without lesion of the frontopolar cortex (main effect of group [F(1,30) = 3.1, P = 0.08]). There was also a main effect of task [F(2,30) = 16.9, P<0.00005] due to the lower error rate in the delay condition. No group*task interaction [F(2,30) = 0.59, P = 0.55] was observed on performance accuracy. When we performed another 2*2 analysis of variance grouping the dual task and multi-tasking conditions together (both involving switching between tasks) compared to the delay condition, we found a trend towards significance between patients with *versus* without frontopolar lesions [F(1,32) = 3.68, P = 0.06]. These data suggest that frontopolar lesions impair switching processes, both compared to controls and compared to patients without frontopolar lesions. No difference between patient group was observed when performing another 2*2 analysis of variance grouping the delay and multi-tasking conditions together (both involving working memory) compared to the dual task [F(1,32) = 0.96, P = 0.33].

### Correlations between performance and damage to the fronto-polar cortex

**Figure 6:**
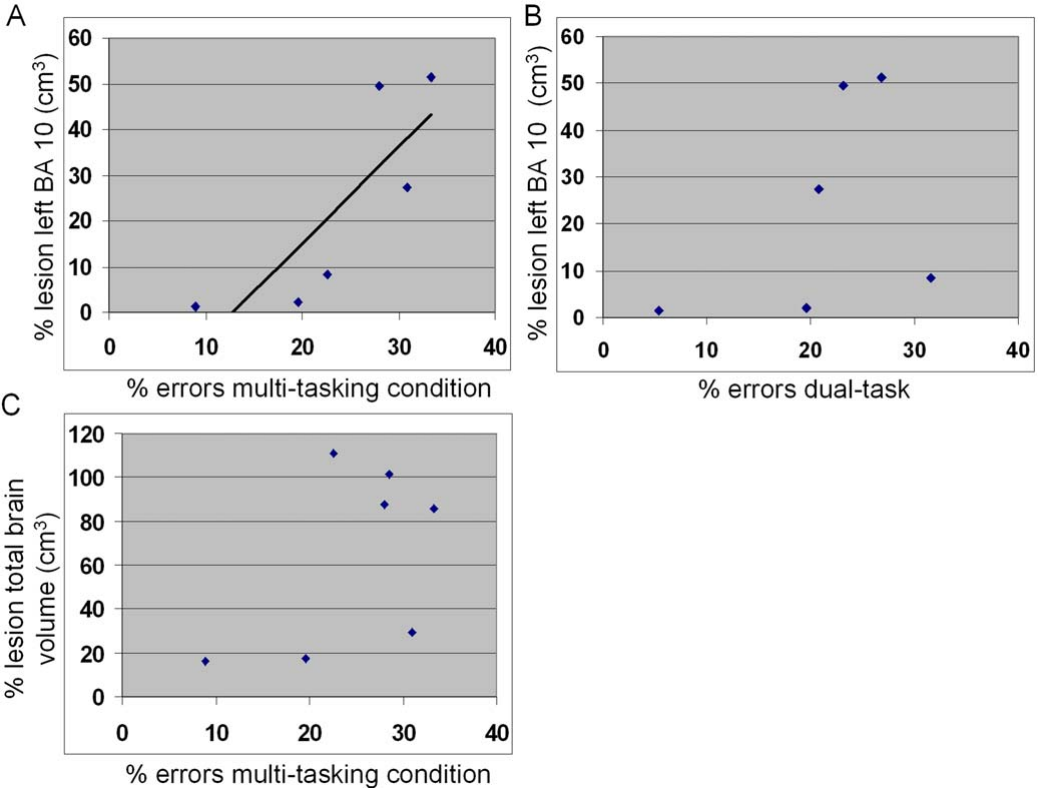
Relationships between lesion size and behavior. (A) A positive significant correlation was observed between the percentage of errors in the multi-tasking condition and the percentage of damage to the left Brodmann's area 10 in patients with fronto-polar cortex lesions (Spearman rank correlation coefficient R = 0.94, P<0.005). (B) No significant correlation was observed between the lesion sizes of the left BA 10 with error rate in the dual-task condition. (C) No significant correlation was observed between total volume of brain lesions and error rate in the multi-tasking condition.

In order to test our specific hypothesis that the fronto-polar cortex (Brodmann's area 10) is necessary for performing a subgoal while maintaining primary goal related information in memory, we correlated the proportion of damage to each approximate Brodmann's area contained within the boundaries of the lesion with the error rates in the multi-tasking condition. Only the left BA 10 (Spearman rank correlation coefficient R_1_ = 0.94, P<0.005) showed a significant positive correlation with performance (6 patients had left frontopolar damage) (**Fig. 6.A**). No other Brodmann's area was significantly correlated with performance in the multi-tasking condition. Moreover, lesion size of the left BA 10 did not show a significant correlation with error rates in the dual-task condition (Spearman rank correlation R_2_ = 0.65, P = 0.16, n.s) (**Fig. 6.B**). This demonstrates that variability of frontopolar cortex patients in multi-tasking performance is primarily explained by the size of the lesion in BA 10.

Note that this analysis did not include the only patient with lesion restricted to the right fronto-polar cortex because there may be a functional lateralization of this brain region. However, when including the data of this subject, the correlation between error rates in the dual-task condition and the size of fronto-polar lesions was still non-significant (R = 0.1, P = 0.5) and the correlation between error rates in the branching condition and size of fronto-polar lesions remained significant (Spearman rank correlation coefficient R = 0.82; P<0.05).

Further analyses of the total volume of brain lesions revealed no relationship between the total volume of damaged tissue (which included extra fronto-polar damage) and error rates in the multi-tasking condition (R = 0.36, P = 0.43), ruling out the possibility of a confound between total lesion size and lesions of the fronto-polar cortex **(** **Fig. 6.C** **)**. Moreover, task difficulty or mental effort alone can not explain our findings since error rates in the multi-tasking and the dual-task condition did not differ significantly in patients with fronto-polar cortex lesions ((F1,19) = 0.4, P = 0.5).

## Discussion

Based on recent neuroimaging findings [3], [4], we could have expected that patients with frontopolar lesions would exhibit a specific increase of errors only in the multi-tasking condition. The fact that we did not observe such a group by task interaction may be due to the extent of the lesions of the frontopolar group, which included, but were not restricted to, the frontopolar region. This may also explain why performance is altered in the dual task condition, thereby masking the behavioral effect of more restricted frontopolar lesions. Note that it is also possible that the frontopolar cortex contains multiple subregions, each contributing to different processes, and/or that brain regions outside the frontopolar cortex are part of a functionally distributed network that is necessary to perform multitasking computations.

Although we did not find a group*task interaction on performance accuracy, likely due to group size and low statistical power, our results suggest that what is crucially impaired in patients with frontopolar lesions is the ability to put information into sequences as required by the dual-task and the multi-tasking conditions rather than simply holding a goal in working memory (such as in the delay condition). Indeed, when performing an analysis of variance grouping the dual task and multi-tasking conditions together (both involving putting information into sequences) compared to the delay condition, we did find a significant difference between patients with frontopolar lesions and controls and a trend towards significance between patients with frontopolar lesions *versus* without frontopolar lesions.

Our main finding is that only the extent of the lesion of the left fronto-polar cortex showed a positive correlation with performance in the multitasking condition. This demonstrates that damage to the frontopolar cortex is necessary to impair multitasking, i.e. a process dependent upon the ability to put tasks in pending sequences. These results provide new evidence that specific executive functions are subserved by distinct prefrontal regions [10], [11], [12], [13], [14], [15], contradicting the view that the functions of distinct prefrontal regions cannot be distinguished [16]. Thus, variability of frontopolar cortex patients in multi-tasking performance is primarily explained by the size of the lesion in BA 10. This correlation between left BA 10 lesion volume and performance is consistent with the linguistic nature of the task. Nevertheless, since there was only one subject with strictly unilateral right frontopolar damage among our subjects, we cannot be certain about whether the effect is truly lateralized.

Although the lesion size of the left BA 10 did not show a significant correlation with error rates in the dual-task condition (Spearman rank correlation R = 0.65, P = 0.16, non significant), it could be argued that the significant correlation observed between lesion size in the fronto-polar cortex and multi-tasking (R = 0.94, p<0.005) does not prove that the effect is specific. However, the inference we tested was that the correlation coefficient was significantly different from zero in the multitasking condition and/or in the dual task condition, not that the correlation in the multitasking condition was significantly higher than the correlation in the dual task. Thus, it would only be statistically justified to compare correlation coefficients (between overlapping pair of variables) if both of these correlations coefficients were significantly different from zero (which is not the case). To further ensure the specificity of our findings, we tested the robustness of our results by recomputing the correlation coefficient in the multitasking and dual-task conditions for each possible n-1 subset of data sample (see **supplementary tables S1 and S2**). These tables show that the significance of the correlation coefficient remains P<0.05 in the multitasking condition and is non-significant in the dual-task condition. This demonstrates that variability of frontopolar cortex patients in multitasking is primarily explained by the size of the lesion in BA 10 and that the size of the left BA 10 lesion is a good anatomical predictor of multitasking but not of dual-task related errors.

Although there may be many functional subregions within the frontopolar cortex, we believe that BA 10 is particularly important for multitasking. This process may be involved in a number of functions previously associated with the frontopolar cortex besides multitasking [17], including integrating the outcomes of two or more separate cognitive operations in the pursuit of a higher behavioural goal [18], processing of internally generated information [19], [20], memory retrieval [21], [22], carrying out delayed intentions (prospective memory) [5], [6], relational integration [23], [24], [25], integration of diverse information content [26] and exploratory decisions [27].

It should be noted that there is a fundamental qualitative difference between multitasking and the dual task. Multitasking combines not only a dual-task component but also a working memory component. It successively allocates processing resources between concurrent tasks, as in dual-task performance and it keeps relevant information in working memory to allow a return to the main task after completing a secondary task. In contrast to multitasking, which specifically involves the fronto-polar cortex, dual task performance induces higher inferior and middle frontal sulcus activity as compared to single task performance [12], [28], [29], [30]. However, our results are not conclusive about other regions than the frontopolar cortex because we did not test patients with specific dorsolateral prefrontal cortex lesions.

A general model, integrating the recent cascade model by Koechlin et al. [31] and the multi-tasking view of the fronto-polar cortex has recently been proposed in a review paper [32]. This general model explains at an information processing level, using information theory, what is called branching (renamed here as multitasking). The overview of this general model is that cognitive control operates according to three nested levels of control processes (contextual, episodic and multi-tasking) implemented from posterior to polar prefrontal regions. In this model, H(a) measures the total amount of control information required for selecting action “a” and is processed in the premotor cortex. H(a) is the sum of two control terms: bottom-up information conveyed by a stimulus S (I(s,a), sensorimotor control) and the remaining top-down information Q(a|s) processed in the posterior lateral PFC and measuring cognitive control. Cognitive control, in turn, is the sum of two control terms: bottom-up information conveyed by the context c in which stimulus s occurs (I(c,a|s), contextual control); and the top-down remaining information Q(a|s,c) processed in the anterior lateral PFC. Finally, this latter control term is the sum of bottom-up information conveyed by a past event u (I(u,a)|s,c), episodic control) and the remaining top-down information processed in the polar lateral PFC (multi-tasking control). Multi-tasking control is related to the information conveyed by events preceding *u* and maintained in a pending state until completion of the ongoing episode. Thus, according to this model, during execution of the current episode *u*, the most anterior portions of the PFC maintain (in a distractor-resistant fashion) pending information from a yet more temporally distant episode, enabling this information to be flexibly retrieved when this episode is re-instantiated. This model explains the pattern of prefrontal activations observed in several experimental paradigms, including learning [33], episodic memory [34], working memory [35] and task switching paradigms [4], [10], [13], [14]. In these experiments, caudal and rostral LPFC activations were observed, depending on whether the executive control of behavior was based on contextual or episodic signals.

A recent review, consistent with our interpretation of frontopolar function, is that a common process across these studies may be that the frontopolar cortex is recruited to integrate the results of two or more cognitive operations, fulfilling a higher behavioural goal [18]. This view predicts that the process of integration should be reflected in frontopolar activity beyond the activity observed for processing the component elements to be integrated. Confirming this prediction, a highly specific super-additive effect was previously demonstrated in the frontopolar cortex using fMRI when subjects held in mind goals while processing secondary goals at the same time [3].

To conclude, we have demonstrated that managing subgoals while maintaining information about primary goals is a process that is critically and selectively disrupted with increasing size of fronto-polar cortex damage. From an evolutionary point of view, it is interesting to note that during hominoid evolution, the frontopolar cortex (area 10) may have undergone not only a shift in its extent but also of its topographic location and a specific increase in connectivity with other higher-order association areas (the supragranular layers having more space available for connections with other higher-order association area) [1], [2]. It has recently been proposed that the frontopolar cortex may be the only prefrontal region that is predominantly (and possibly exclusively) interconnected with supramodal areas in the prefrontal cortex and anterior temporal cortex [18], allowing the frontopolar cortex to dynamically monitor and assign positional priority to information received from more caudal areas of supramodal cortex. Since the frontopolar cortex in the human brain appears to have evolved in size and organization, this suggests that complex functions requiring the temporary interruption of a current plan to achieve subgoals (such as planning of future actions and reasoning) associated with this part of the cortex have become particularly important during hominid evolution.

## Supporting Information

Table S1 Correlation coefficient in the branching condition for each possible n-1 subset of data sample.
(0.03 MB DOC)

Table S2 Correlation coefficient in the dual-task condition for each possible n-1 subset of data sample.
(0.02 MB DOC)
