# Supplementary material for: Damage to the Fronto-Polar Cortex Is Associated with Impaired Multitasking
Source: PLoS One. 2008 Sep 16;3(9):e3227. doi: 10.1371/journal.pone.0003227 (PMC2528949; doi:10.1371/journal.pone.0003227)
Supplement: Table S1 — Correlation coefficient in the branching condition for each possible n-1 subset of data sample. (0.03 MB DOC) [file pone.0003227.s001.doc]

**Supplementary table 1.**

| BRANCHING correlation | Spearman R | P |
| --- | --- | --- |
| All points | 0.94 | <0.05 |
| Without point 1 | 0.9 | <0.05 |
| Without point 2 | 0.9 | <0.05 |
| Without point 3 | 0.9 | <0.05 |
| Without point 4 | 1 | <0.05 |
| Without point 5 | 1 | <0.05 |
| Without point 6 | 0.9 | <0.05 |
