# Supplementary material for: Damage to the Fronto-Polar Cortex Is Associated with Impaired Multitasking
Source: PLoS One. 2008 Sep 16;3(9):e3227. doi: 10.1371/journal.pone.0003227 (PMC2528949; doi:10.1371/journal.pone.0003227)
Supplement: Table S2 — Correlation coefficient in the dual-task condition for each possible n-1 subset of data sample. (0.02 MB DOC) [file pone.0003227.s002.doc]

**Supplementary table 2.**

| DUAL TASK correlation | Spearman R | P |
| --- | --- | --- |
| All points | 0.65 | n.s |
| Without point 1 | 0.7 | n.s. |
| Without point 2 | 0.6 | n.s. |
| Without point 3 | 0.4 | n.s. |
| Without point 4 | 0.7 | n.s. |
| Without point 5 | 0.7 | n.s. |
| Without point 6 | 0.4 | n.s. |
